# A Specific and Rapid Neural Signature for Parental Instinct

**DOI:** 10.1371/journal.pone.0001664

**Published:** 2008-02-27

**Authors:** Morten L. Kringelbach, Annukka Lehtonen, Sarah Squire, Allison G. Harvey, Michelle G. Craske, Ian E. Holliday, Alexander L. Green, Tipu Z. Aziz, Peter C. Hansen, Piers L. Cornelissen, Alan Stein

**Affiliations:** 1 Department of Psychiatry, University of Oxford, Oxford, United Kingdom; 2 Department of Physiology, Anatomy and Genetics, University of Oxford, Oxford, United Kingdom; 3 Department of Psychology, University of California, Berkeley, California, United States of America; 4 Department of Psychology, University of California Los Angeles, Los Angeles, California, United States of America; 5 The Wellcome Trust Laboratory for MEG Studies, School of Life and Health Sciences, Aston University, Birmingham, United Kingdom; 6 School of Psychology, University of Birmingham, Birmingham, United Kingdom; 7 Department of Psychology, York University, York, United Kingdom; 8 Department of Neurosurgery, John Radcliffe Hospital, Oxford, United Kingdom; 9 Center of Functionally Integrative Neuroscience (CFIN), Aarhus University, Aarhus, Denmark; University of St. Andrews, United Kingdom

## Abstract

Darwin originally pointed out that there is something about infants which prompts adults to respond to and care for them, in order to increase individual fitness, i.e. reproductive success, via increased survivorship of one's own offspring. Lorenz proposed that it is the specific structure of the infant face that serves to elicit these parental responses, but the biological basis for this remains elusive. Here, we investigated whether adults show specific brain responses to unfamiliar infant faces compared to adult faces, where the infant and adult faces had been carefully matched across the two groups for emotional valence and arousal, as well as size and luminosity. The faces also matched closely in terms of attractiveness. Using magnetoencephalography (MEG) in adults, we found that highly specific brain activity occurred within a seventh of a second in response to unfamiliar infant faces but not to adult faces. This activity occurred in the medial orbitofrontal cortex (mOFC), an area implicated in reward behaviour, suggesting for the first time a neural basis for this vital evolutionary process. We found a peak in activity first in mOFC and then in the right fusiform face area (FFA). In mOFC the first significant peak (p<0.001) in differences in power between infant and adult faces was found at around 130 ms in the 10–15 Hz band. These early differences were not found in the FFA. In contrast, differences in power were found later, at around 165 ms, in a different band (20–25 Hz) in the right FFA, suggesting a feedback effect from mOFC. These findings provide evidence in humans of a potential brain basis for the “innate releasing mechanisms” described by Lorenz for affection and nurturing of young infants. This has potentially important clinical applications in relation to postnatal depression, and could provide opportunities for early identification of families at risk.

## Introduction

Darwin originally pointed out that there is something about infants which prompts adults to respond to and care for them, in order to increase individual fitness, i.e. reproductive success, via increased survivorship of one's own offspring [1]. Konrad Lorenz argued that infantile features serve as a *Kindchenschema* (infant schema) [2] with “innate releasing mechanisms” for affection and nurturing in adult humans and that most of these features are evident in the face including a relatively large head, predominance of the brain capsule, large and low lying eyes and bulging cheek region [3]. It is argued that these “babyish” features increase the infant's chance of survival by evoking parental responses [4], [5]. However, the neural basis for the responses to infant face compared to that of adult faces has not yet been elucidated.

Face processing in general has been studied extensively in macaques, with single neuron recordings and neuroimaging using fMRI [6], [7]. Also, human fMRI experiments have found activity specific to faces in an area of right posterior fusiform cortex corresponding to the fusiform face area (FFA) [8], [9]. MEG studies using dipole modelling found early focal occipotemporal activity to faces relative to words [10]. It is still, however, controversial whether the activity in FFA represents a localized or distributed coding of faces [8], [11], [12].

Processing of infant faces may be distinctly different because of its importance in eliciting parental responsivity and care. A number of studies have used fMRI to examine parental responses to infant faces [13]. Most compare parental responses to their own infants and other infants, and show stronger activity to own infants in striate and extrastriate visual areas and in reward-related areas such as the nucleus accumbens, anterior cingulate and amygdala [13], [14]. However, these studies do not test whether there is something special about infant faces per se rather than one's own infant's face partly because this type of comparison is likely to be confounded by familiarity.

A substantial test of Lorenz's theory of the specificity of infant faces requires a direct comparison between matched adult faces and infant faces from the first year of life; preferably using unfamiliar faces and neuroimaging techniques that permit the temporal progression of brain activity to be studied. Yet, no published studies have compared responses to unfamiliar infant faces with responses to unfamiliar adult faces.

We hypothesized that adult humans would show distinct brain responses to unfamiliar infant faces compared to adult faces and that these differences in brain activity might potentially be found relatively early in time. We therefore chose MEG to measure changes in brain activity over milliseconds. In order to measure the distinctiveness of the features of the infant face compared to the adult face, we used multiple faces of real infants and adults with the each face shown with positive (smiling), neutral and negative (sad) expressions. We matched the face stimuli as closely as possible across the two groups of unfamiliar infant and adult faces, according to emotional valence and arousal, as well as size and luminosity in order to exclude these potential confounds. The faces also matched closely in terms of attractiveness. (see Methods). We were particularly interested in the role of the orbitofrontal cortex, which has previously been implicated in reward and hedonic processing [15]. Furthermore, prefrontal areas such as the orbitofrontal cortex have been shown to perform a top-down facilitation of the areas involved in visual object recognition in the fusiform cortices [16].

## Results

**Figure 1:**
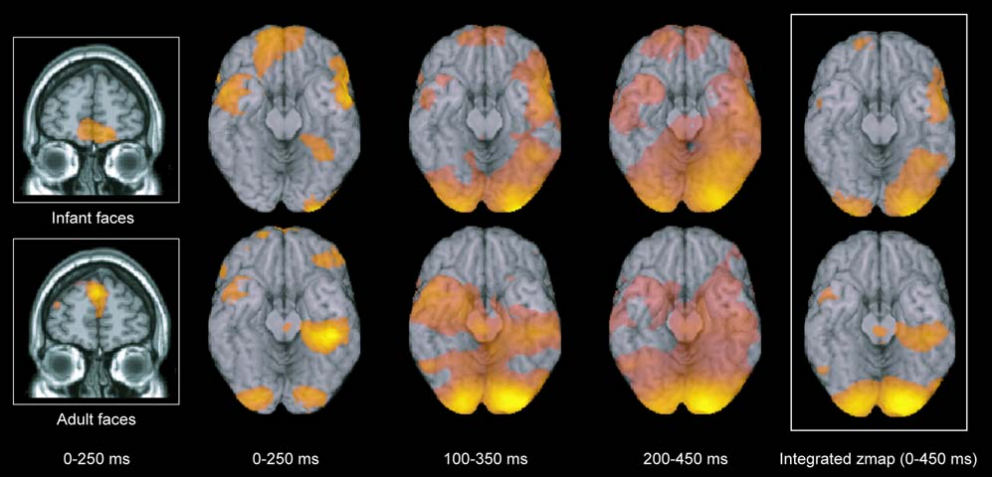
Significant differences between viewing infant and adult faces. The group SAM analysis revealed a significant peak in the medial orbitofrontal cortex in the 10–30 Hz band in the 0–250 ms (first two columns), 100–350 ms (third column) and 200–450 ms (fourth column) windows when participants viewed infant (upper row) and not when they viewed adult faces (lower row). The fifth column shows the integrated z-map over the three time windows (with Z>3.1) with all active brain regions listed in Table 1. In order to see the extent of the spread of activity over the fusiform cortices elicited by faces, the group activity is superimposed on a ventral view of the human brain (with the cerebellum removed).

Consistent with previous findings [17], we found that face processing of both adult and infant faces elicits similar waves of activity starting in the striate cortices and spreading along ventral and dorsal pathways (see **Figure 1** **and** **Table 1**).

**Table 1:** Active brain regions.

*Brain region*	Infant faces					Adult faces				
	*l*	*x*	*y*	*z*	*z-stat*	*l*	*x*	*y*	*z*	*z-stat*
Occipital pole/lateral occipital cortex	R	38	−96	12	6.7	R	20	−90	−6	6.2
	L	−2	−102	−16	5.4	L	−8	−94	−18	5.7
Middle temporal gyrus/temporal pole	R	60	4	−32	4.5	L	−56	8	−36	3.0
	L	−60	2	−36	3.2	L	−18	6	−36	2.9
Fusiform cortex	R	44	−52	−24	4.1	R	44	−28	−20	4.1
Postcentral gyrus/supramarginal gyrus	L	−28	−42	64	3.7	L	−50	−24	62	3.6
	R	68	−14	16	3.1	L	−4	−54	76	3.2
	L	−54	−26	32	3.5					
Precentral gyrus/middle frontal gyrus	L	−40	−4	64	3.7	L	−58	14	36	2.7
										
Inferior frontal gyrus, pars triangularis	R	48	26	10	3.5	L	−58	26	12	2.7
										
**Medial orbitofrontal cortex**	**L**	**−22**	**62**	**−16**	**3.5**					**n/s**
Superior frontal gyrus/supplemental motor area	L	−6	0	74	3.5	L	−28	30	54	3.4
	R	26	0	58	3.3					
Lateral occipital cortex	L	−58	−60	34	3.4	R	46	−82	30	4.6
Frontal pole	L	−28	52	42	3.2	R	38	50	32	3.3
						L	−2	60	30	3.3
Cerebellum	L	−12	−56	−44	3.2	L	−58	−62	−26	3.3

*Active regions are significant at Z>2.7, except for the activity in the medial orbitofrontal cortex which was not significant (n/s) at this threshold. All reported brain coordinates are in the standard space of MNI (Montreal Neurological Institute).*

List of active brain regions (from the integrated z-map in figure 1) in an implicit task attending a change in colour of a fixation during which infant and adult faces were presented for 300 ms but, crucially, did not help complete the task. As expected, both infant and adult faces elicited significant activity mainly in striate cortices and along ventral and dorsal pathways. Importantly, however, there was significant activity in the medial orbitofrontal cortex only to the infant faces but not to the adult faces.

In addition to this early activity in these visual areas, the principal finding of the group SAM analysis was more significant activity in the medial orbitofrontal cortex in the 10–30 Hz band in the three time windows of 0–250 ms, 100–350 ms and 200–450 ms as well as in the integrated z-map, when participants were viewing the infant faces but *not* when they were viewing the adult faces (Z>3.5, p<0.001) (see **Figure 1** and **Table 1**). We found the same result in the medial orbitofrontal cortex using both scanners; that is, in the initial four participants and again in the eight participants scanned on the more powerful MEG scanner. The complete set of regions active in the integrated zmap over the three time windows thresholded at Z>3.1 are shown in **Table 1**.

**Figure 2:**
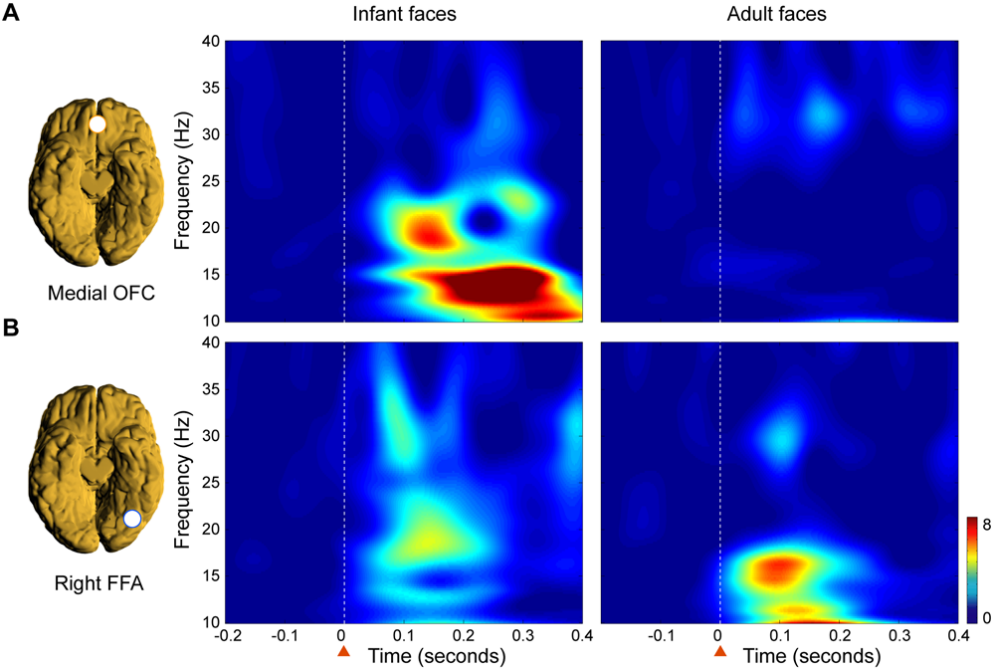
Time-frequency analysis of neural activity in medial orbitofrontal cortex (OFC) and the right fusiform face area (FFA). Significantly different responses were found in the medial OFC but not in the right FFA between viewing infant compared to adult faces. A) Time-frequency representations of the normalised evoked average group responses to infant and adult faces from the virtual electrodes in the medial OFC reveal that the initial response to infant faces is present in the 12–20 Hz band from around 130 ms-and not present to adult faces. B) The responses in right FFA occurred earlier in time but were not significantly different before 165 ms when viewing infant compared to adult faces. This can be seen from the time-frequency representations of the normalised evoked average group from the virtual electrodes, where initial activity was present from around 100 ms in the 10–20 Hz and in the 25–35 Hz bands. The white stippled line and the orange arrow indicates when the faces were presented in time.

We carried out further detailed group time-frequency analyses to characterize the nature of the response in the medial orbitofrontal cortex and in the right fusiform face area (see methods and **Figure 2**). At around 130 ms after presentation of a face, significantly more activity was found in the medial orbitofrontal cortex in response to infant than to adult faces in the 10–15 Hz band (p<0.001) (see **Figure 2A**). This striking difference in activity elicited by infant compared to adult faces was *not* found in the right fusiform face area, where the initial activity occurred earlier around 100 ms in the 10–20 Hz and in the 25–35 Hz bands (see **Figure 2B**).

**Figure 3:**
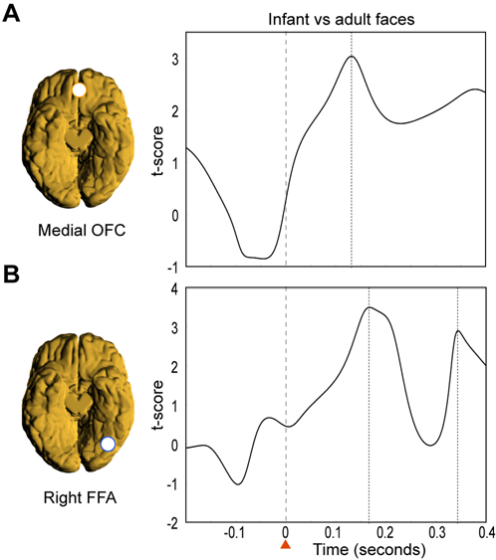
Comparing the power changes in activity for infant vs adult faces. Significant differences in power changes in activity were found first in the medial OFC and then in the right FFA. A) In the medial OFC the first significant peak (p<0.001) in differences in power between infant and adult faces in the 10–15 Hz band was found at around 130 ms. These early differences were not found in the FFA. B) In contrast, differences in power were found later, at around 165 ms, in a different band (20–25 Hz) in the right FFA.

To evaluate the sequence of stimulus elicited activity in the FFA and OFC, we compared the power changes in activity in both regions. We found a peak in activity first in the medial orbitofrontal cortex and then in the right FFA. In the medial orbitofrontal cortex the first significant peak (p<0.001) in differences in power between infant and adult faces in the 10–15 Hz band was found at around 130 ms (see **Figure 3A**). These early differences were not found in the FFA. In contrast, differences in power were found later, at around 165 ms, in a different band (20–25 Hz) in the right FFA (see **Figure 3B**).

To test whether these results held when restricted to participants who were not parents, we excluded the three parents from the analysis. Using solely the data from the non-parents produced the same results.

## Discussion

The principal finding was a relatively brief surge in activity in the medial orbitofrontal cortex at around 130 ms in response to infant faces but not in response to adult faces. In contrast, in the early visual regions including the FFA, face processing of infant and adult faces followed a similar pattern. Thus, the medial orbitofrontal cortex appears to exhibit a very early specific neural signature or specific pattern of activity in response to infant faces. This signature is likely to be directly related to saliency of the structural features of the infant face rather than to other factors, since the infant and adult faces were carefully matched in terms of emotional valence and arousal, and attractiveness (see Methods). Supporting this, the signature, which is well-characterised both temporally and spatially, is not found in the right fusiform face area and other early visual areas, where both infant and adult faces instead elicit very similar neural responses.

The early specific surge of activity in the medial orbitofrontal cortex to infant faces at around 130 ms in the 10–15 Hz band (**Figure 2A** **and** **3A**) was then followed by an enhanced response at around 165 ms in the FFA in the 20–25 Hz band (**Figure 2B** **and** **3B**). This suggests that the medial orbitofrontal cortex provides a top-down amplification of the activity in the FFA specifically related to infant faces.

This finding extends previous research which has shown that early orbitofrontal cortex activity facilitates visual recognition of *masked* line drawings of everyday objects [16]. In this task, Bar et al. tested the subjective certainty of visual object recognition of very briefly presented line drawings (63 ms) which were preceded and followed by visual masks. During these brief presentations the authors found early activity in the orbitofrontal cortex, which was not, however, evident when the same line drawings were presented non-masked for a longer stimulus duration (198 ms). The effect would thus seem to be task specific in that it was only present on those trials where participants were instructed to indicate their level of knowledge about the identity of the object. The early activity in medial orbitofrontal cortex is therefore likely to be closely linked to the salience or attentional processing of the masked stimuli. In the context of the present experiment, where we have demonstrated that early activity in the medial orbitofrontal cortex is linked to the presentation of salient *non-masked* infant faces but not to adult faces, these findings would suggest that the structural configuration of infant faces might act as a heightened attentional/emotional biasing mechanism, consistent with the recent behavioural findings using infant faces in a dot probe attentional paradigm [18].

The medial orbitofrontal cortex may thus provide the necessary attentional–and perhaps emotional–tagging of infant faces that predisposes humans to treat infant faces as special and elicits caring, as suggested by Darwin [1] and Lorenz [2].

Since the infant and adult faces used in the present study were carefully matched by independent panels of participants for emotional valence and arousal, and attractiveness (see Methods), the present findings provide evidence that it is the distinct features of the infant faces compared to adult faces which are important, rather than evaluative subjective processing such as attractiveness or emotional valence.

A number of functional MRI studies have found a correlation between facial attractiveness and changes in the BOLD signal in the medial orbitofrontal cortex [19], [20]. This was interpreted as indicating that the medial orbitofrontal cortex has a specific role in attractiveness. However, there are also several fMRI studies, which have found that the BOLD signal in the medial orbitofrontal cortex is correlated with the subjective ratings of many different stimuli in different modalities such as olfaction [21], gustation [22], somatosensory [23] and multimodal [24]. It has therefore been argued that these findings indicate a role for the medial orbitofrontal cortex in the monitoring, learning and memory of salient reward-related stimuli in the environment–rather than attractiveness per se [15].

Furthermore, all these fMRI findings are concerned with ongoing evaluative changes in activity in the medial orbitofrontal cortex which occur both *later* in time and *over longer periods*, compared to the early transient burst of activity to infant faces at around 130 msec found with MEG in the present study. The present findings are therefore potentially of interest in that they suggest a temporally earlier role than previously thought for the medial orbitofrontal cortex in guiding affective reactions, which may be even non-conscious. This processing could be an important foundation for subsequent integrative and evaluative subjective processing.

The suggested monitoring process of the medial orbitofrontal cortex is consistent with intriguing findings in spontaneously confabulating patients with lesions to the medial orbitofrontal cortex [25]. The evidence from human neuroimaging and neuropsychology studies suggests that there are medial-lateral and posterior-anterior distinctions within the human orbitofrontal cortex [26]. This meta-analysis of the existing neuroimaging data showed that activity in the medial orbitofrontal cortex is related to the monitoring, learning and memory of the reward value of reinforcers, whereas lateral orbitofrontal cortex activity is related to the evaluation of punishers, which can lead to a change in ongoing behaviour.

Notably, the results did not differ when restricted to the non-parent portion of the sample. However, further investigation is required into a number of areas including the relevance of parental experience, gender, and valence of emotional expression (whether brain responses differ between positive and negative emotions) and whether there are specific brain responses to individual infant features such as large eyes. In addition, given the evidence from various behavioral studies showing that many cute infantile things appear to invoke Lorenz's “innate releasing mechanisms” [27], [28]. It might also be of interest to future studies to investigate the brain responses to infants of other species.

There is a potentially important clinical application of the present findings in relation to postnatal depression. Postnatal depression is common, occurring in approximately 13% of mothers after birth [29] and often within six weeks [30]. Postnatal depression has been associated with a range of adverse child outcomes including attachment, behavioural and emotional disturbances and there is also some evidence for poorer cognitive outcomes. There is increasing evidence that certain features of the behaviour of depressed mothers are associated with adverse outcome, in particular their lack of responsiveness to the infant, the reduced ability to perceive their infant's signals and less mimetic behavior [31], [32] with a resultant lack of contingency between the infants actions and the mothers responses [33]. Furthermore, it has been shown experimentally that infants respond adversely with distress, crying, increased arousal and then avoidance to an unresponsive maternal face (the still face paradigm) [31], 34. Also, there is now evidence from deep brain stimulation linking depression to the nearby subgenual cingulate cortex which is strongly connected with the medial orbitofrontal cortex [35]. This lends support to the possibility that changes to activity in the medial orbitofrontal cortex secondary to depression may adversely affect parental responsivity. Further research could identify whether these early and specific medial orbitofrontal responses to infant faces (own and others) are affected and even suppressed by depression, thereby helping to explain this lack of maternal responsiveness. The present paradigm could eventually provide opportunities for early identification of families at risk [13].

To conclude, we found a very specific, rapid neural signature of activity in the medial orbitofrontal cortex in response to infant faces. This provides evidence in humans of a potential brain basis for the “innate releasing mechanisms” described by Lorenz for affection and nurturing of young infants. Although the degree to which these responses are innate rather than learned is unknown; at the very least, the specific responses to unfamiliar infant faces in the medial orbitofrontal cortex occur so quickly that they are almost certainly quicker than anything under conscious control.

## Materials and Methods

### Behavioural methods

Infant and adult faces were taken from two databases and matched for attractiveness, emotional expression and arousal. The infant faces were taken from a database of digital photographs of infant facial expressions that was produced from digital videotapes of 27 infants (aged 3 m–12 m), who were filmed in their own homes with approval from the Oxford Research Ethics Committee. The database is unique in that it contains pictures of the *same* infants expressing positive, negative and neutral emotions, controlling for head direction and eye gaze, as far as possible. Each parent gave permission for the children's faces to be used in this task. The adult faces were taken from the Ekman database of faces [36].

Ninety-five adults (25 male, 70 female; mean age 19 years 10 months) rated the infant faces; each face was rated by 30 adults. Ratings were made on dimensions of valence and arousal (via the Self-Assessment-Manikin, as used for validating the International Affective Picture System [37]) and each emotional expression was rated from –4 (very negative affect) to +4 (very positive affect). The same observers also rated a selection of Ekman adult faces on the same scales. We were careful to select the happy, neutral and sad faces of 13 infants and 13 adults such that the stimuli were matched for emotional valence and arousal (infants: 0.2 (1.9), mean (standard deviation) and adults: −0.1(2.1), not significant) across the two groups. The matched ratings for happy, neutral and sad infants and adults were as follows: happy faces (2.7(0.4) mean (s.d.), vs 2.6(0.5), not significant); neutral faces (–0.3(0.6) mean (s.d.) vs –0.7(0.9), non-significant); sad faces (–1.8(0.5) mean (s.d.) vs –2.2(0.7), not significant).

All face images used in the imaging task were presented as grayscale images, with adult and infant faces matched for emotional expression, as well as size and luminosity to minimize possible confounding effects of visual appearance. Participants viewed the faces on a computer monitor, such that face stimuli subtended a visual angle of approximately 4×2°.

### Task and imaging methods

MEG was chosen because it enables measurement of the full spatiotemporal evolution in adult brain activity, and the detection of very early responses (within 150 msecs) which is the time period within which most visual perception occurs [38], [39].

Each participant gave written consent to participate in the MEG study in accordance with the principles of the Helsinki Declaration. They then performed an implicit task in the MEG scanner. During the task, a fixation cross was on the screen at all times except when replaced by blocks of either happy, neutral or sad adult and infant faces for 300msecs. Attentional load was balanced by requiring participants to maintain fixation at all times on the small red fixation cross, to ignore the faces, and to press a button whenever the fixation cross changed colour to green. These events occurred pseudo-randomly with an average frequency of one colour change per 16 visual object presentations. These response trials were subsequently discarded from the data analysis to ensure that the MEG signal was not contaminated by motor responses. Subsequent to performing the task in the scanner, we also had the participants rate the neutral faces for attractiveness from −4 (not very attractive) to +4 (very attractive) to make sure this was not a potential confound (infants: 0.1(0.9), mean (s.d.) vs adults: 0.0(1.2), non-significant).

### Imaging methods

Twelve participants (seven women and five men, nine were not parents, overall mean age: 29.5 years) were scanned using MEG. The MEG data was collected using two different MEG systems. The first four participants were scanned on a 151-channel CTF Omega system (CTF Systems Inc., Port Coquitlam, Canada) at Aston University. Data were sampled at 1250 Hz with an antialiasing cut-off filter of 200 Hz. The further eight participants were scanned using a 275-channel CTF Omega system (CTF Systems Inc., Port Coquitlam, Canada) at Aston University. These data were sampled at a higher sampling frequency of 2500 Hz with an antialiasing cut-off filter of 200 Hz. All participants were also scanned with MRI to get a high resolution T1 volume with at least 1×1×1 mm voxel dimensions. Immediately after finishing data acquisition, a 3-D digitizer (Polhemus Fastrack, Polhemus Corporation, Colchester, VT, US) was used to digitize the shape of the participant's head in the MEG laboratory and the relative position of the headcoils for the nasion, left and right ear on the headset, which was then matched to the participant's MRI.

### Analysis method

We used SAM to generate statistical parametric maps of stimulus related changes in cortical oscillatory power. SAM is a non-linear beamformer linking each voxel in the brain with the MEG sensors by constructing an optimum spatial filter for that location [40], [41]. This spatial filter is a set of weights and the source strength at the target location is computed as the weighted sum of all the MEG sensors. This output is also called a ‘virtual electrode’ which has the same millisecond temporal resolution as the initial MEG recordings [42].

In this experiment, the SAM analysis created a volume for covering the whole brain in each individual with a voxel size of 5×5×5 mm. The passive state was defined as the time period between −250 and 0 ms before stimulus onset and the active state was defined as a moving time window starting at 0 ms before stimulus onset to 250 ms after the onset. Power changes between the active and passive states were calculated for the beta (10–30 Hz) and gamma (30–60 Hz) frequency bands. Furthermore, in the data analysis we took care to eliminate eyeblink artefacts.

Group statistical maps for each frequency band were generated by first normalising the SAM functional volumes to MNI standard space [43] and then combining these volumes across participants for each time window and frequency band. The normalisation parameters were obtained using FLIRT (FMRIB's Linear Image Registration Tool) [44] to reslice each individual's anatomical MRI to the same orientation and position as the SAM functional volume and finding the transformation matrix from this functional space into the standard MNI space. This transformation matrix was then applied to each of the functional SAM volumes, in each time window and frequency band, and for each participant. A simplified mixed-effects model was used to generate group statistical maps by combining volumes across individuals for each contrast by calculating the sum of individual statistical values divided by the square root of the number of participants over each voxel in the standard brain [45]. Further, an integrated group model across time was generated over the time windows by calculating the sum of the t-scores from the groupmap of each timewindow and dividing by the square root of the number of time windows [45].

Detailed time-frequency representations (TFRs) were calculated in areas showing differences between adult and infant faces as well as the FFA. The average time courses in the virtual electrodes were calculated as follows. The first step in virtual electrode analysis required identification of which frequency bands in the SAM analysis gave the strongest signal across the region of interest in the medial orbitofrontal cortex and right fusiform face area from which virtual electrodes would be selected. We extracted the SAM beamformer weights from the statistical peak values in the orbitofrontal cortex and in the right fusiform face area in each individual participant scanned with the 275-channel MEG scanner. Subsequent TFR analysis was performed using the FieldTrip toolbox developed at the F. C. Donders Centre for Cognitive Neuroimaging (http://www.ru.nl/fcdonders/fieldtrip) using Matlab 6.5 (MathWorks, Natick, MA). The TFRs were obtained using a wavelet transform according to the procedures of Tallon-Baudry et al. [46]. The TFRs of power were generated by averaging the squared absolute values of the convolutions over trials for a given condition (i.e., when presenting infant faces or adult faces). Group averages for each condition were generated by normalizing and averaging across participants. The average TFRs of the responses to infant and adult faces at the peak in power in each individual subject were then directly compared using student t-tests which were then taken to the group level.
